# Transcriptomic Analysis of Degradative Pathways for Azo Dye Acid Blue 113 in *Sphingomonas melonis* B-2 from the Dye Wastewater Treatment Process

**DOI:** 10.3390/microorganisms10020438

**Published:** 2022-02-14

**Authors:** Aalfin-Emmanuel Santhanarajan, Chaeyoung Rhee, Woo Jun Sul, Keunje Yoo, Hoon Je Seong, Hong-Gi Kim, Sung-Cheol Koh

**Affiliations:** 1 Department of Environmental Engineering, Korea Maritime and Ocean University, Yeongdo-gu, Busan 49112, Korea; eaalfi@gmail.com (A.-E.S.); kjyoo@kmou.ac.kr (K.Y.); 2 Department of Energy Engineering, Future Convergence Technology Research Institute, Gyeongsang National University, 501 Jinju-daero, Jinju 52828, Korea; chaeyoung415@gmail.com; 3 Department of Systems Biotechnology, Chung-Ang University, Anseong 06974, Korea; sulwj@cau.ac.kr (W.J.S.); hoonjeseong@gmail.com (H.J.S.); 4 Bayo Inc., Jinju 52665, Korea; hg-kim62@hanmail.net

**Keywords:** azo dye, Acid Blue 113, decolorization, biodegradation, dye wastewater treatment

## Abstract

Background: Acid Blue 113 (AB113) is a typical azo dye, and the resulting wastewater is toxic and difficult to remove. Methods: The experimental culture was set up for the biodegradation of the azo dye AB113, and the cell growth and dye decolorization were monitored. Transcriptome sequencing was performed in the presence and absence of AB113 treatment. The key pathways and enzymes involved in AB113 degradation were found through pathway analysis and enrichment software (GO, EggNog and KEGG). Results: *S. melonis* B-2 achieved more than 80% decolorization within 24 h (50 and 100 mg/L dye). There was a positive relationship between cell growth and the azo dye degradation rate. The expression level of enzymes involved in benzoate and naphthalene degradation pathways (NADH quinone oxidoreductase, N-acetyltransferase and aromatic ring-hydroxylating dioxygenase) increased significantly after the treatment of AB113. Conclusions: Benzoate and naphthalene degradation pathways were the key pathways for AB113 degradation. NADH quinone oxidoreductase, N-acetyltransferase, aromatic ring-hydroxylating dioxygenase and CYP450 were the key enzymes for AB113 degradation. This study provides evidence for the process of AB113 biodegradation at the molecular and biochemical level that will be useful in monitoring the dye wastewater treatment process at the full-scale treatment.

## 1. Introduction

Different classes of dyes are used in numerous industries, including the rubber, textile, cosmetic, plastic, leather, food, and paper manufacturing industries. Various dyes are seen in the wastewater discharged from these industries [1]. Among these, the largest contributor to dye wastewater is the textile industry, which is responsible for two-thirds of the total production of dye wastes due to the high quantities of water used in the dyeing processes [2,3,4,5]. The major problem is the unfixed dyes that remain in the wastewater after textile processing [5]. During dyeing processes, approximately 15–30% of the dyestuff fails to bind to the fibers and is therefore released into the environment [6].

Dyes are usually recalcitrant and may be toxic to organisms [7]. Therefore, the release of wastewater containing dyes is quite harmful to the environment. Their presence in an aquatic ecosystem reduces the penetration of sunlight to benthic organisms, thus limiting the process of photosynthesis [1]. Furthermore, dyes reduce the solubility of oxygen in the water. Dyes also affect the aesthetic value of an aquatic ecosystem due to the coloration of water resources. The key concern for the wastewater treatment is the environmental release of dyes and their metabolites that may be mutagens and carcinogens [8].

Azo dyes, as the largest group of synthetic dyes, constitute up to 70% of all the known commercial dyes produced [9,10]. Their chemical structure is characterized by highly substituted aromatic rings joined by one or more azo groups (-N=N-) [7]. This double bond structure is a chromophore that makes the color visible, and thus cleavage of the azo bond will eventually decolorize the dye. Furthermore, an azo bond breaks by liberating the aromatic amine, which is comparatively more toxic than the parent dye [11] and therefore, it is desirable to degrade and remove the dyes in an eco-friendly manner—one that will affect neither the environment nor human health [9]. In particular, azo dyes are regarded as xenobiotic in nature and recalcitrant to biodegradation [12]. For this reason, Acid Blue 113 (AB113), one of the azo dyes, was targeted for its degradation process in this study.

Azo reactive dyes, such as Acid blue 113 (AB113), contribute about 50% to the total market and are widely used in textile industry due to their high permanence [13]. As dyes are designed to be stable in chemical and photolytic ways, they are highly tenacious in a natural environment and may be hard to degrade towards biological wastewater treatment systems due to the complexity of their chemical structure. Around 15% of these dyes are usually lost in the effluent during synthesis and dyeing process [9]. Each azo group is linked to two carbon atoms, which are part of naphthalene and benzene derivatives [14]. In addition, the aromatic rings in these dyes are toxic and cause a major problem in wastewater treatment [15]. In the understanding of the degradation of AB113, the most studied aspects are chemical catalysis and physical adsorption. The chemical and physical methods work well, but they both have some drawbacks in that these processes are much expensive and generate amine residues contained in sludge after degradation. Moreover, regular consumption of such untreated or poorly treated toxic waters shows to be carcinogenesis in humans [16,17]. For AB113, the degradation occurs through the cleavage of the azo double bond (N=N) and the degradation of the aromatic ring. However, none of these reports thoroughly studied the specific mechanisms of the dye degradation.

The discoloration effect of *Pseudomonas stutzeri* AK6 on textile wastewater only reached 86.2% after 96 h [18]. Previous research reported that in the biological decolorization of azo dyes, microorganisms require a carbon source as they are unable to use the azo compounds as the sole carbon source. Nevertheless, excessive amounts of carbon can reduce the decolorization rate as microorganisms prefer to consume external carbon sources rather than dyes. FT-IR spectra analysis enables the determination and understanding of the various functional groups involved in the process of biotransformation [19,20].

Chemical and physical treatment technologies have been well developed and are widely used to treat dye wastewater [21]. However, these approaches have not been economically feasible due to the low rate of color removal, high cost, the production of a large amount of sludge, ineffectiveness in chemical oxygen demand (COD) removal, and production of secondary wastes [3,8,12]. In contrast, the use of microbial treatment technologies has advantages in treating azo dye wastewater because they are environmentally friendly, cost competitive, and can produce less sludge and yield end products that are non-toxic or can mineralize the target chemicals and require less water consumption compared to physicochemical methods [22].

Therefore, bioaugmentation, a biological treatment technology, has the potential to sustainably and efficiently bioremediate dye wastewater [4]. The addition of specific microorganisms has been shown to be more effective than using a consortium of non-specific functions because they are selectively designed for individual degradative bioprocesses [23,24,25]. Bacterial and yeast decolorization methods are widely used due to their high activity and adaptability [26].

Various types of enzymes, including bacterial azoreductases, lignin peroxidase (LiP), manganese peroxidase (MnP), and laccase (Lac) from yeast, are able to effectively degrade xenobiotics such as azocompounds [27]. However, decolorization intermediates, such as aromatic amines, can inhibit the biodegradation activity of bacteria [28]. Diverse organics can be degraded by bacteria, and they are adaptable to a wide range of environments, since most bacteria lack the metabolic enzymes needed to degrade decolorization intermediates (especially aromatic amines) [10]. According to previous research, *Sphingomonas* sp. has certain decolorization and degradation abilities in dye wastewater [29,30,31]. However, there is not much clear information about the peer molecular understanding of the degradation of azo dyes by *Sphingimonas melonis*.

In this study, we selected one of the most appropriate cultures (*S. melonis* B-2) for the efficient bioremediation of azo dye wastewater. HiSeq high-throughput sequencing technology was used to study differential changes in the transcription level of *S. melonis* B-2 undergoing azo dye biodegradation. The specific genes encoding enzymes of *S. melonis* B-2 involved in the degradation were elucidated and selected as potential biomarkers that would be useful in monitoring the full-scale dye wastewater treatment process.

## 2. Materials and Methods

### 2.1. Isolation of Microorganisms Efficient for Azo Dye Biodegradation and Their Identification

A commercial microbial consortium product (CES-1) [24,25] from Bayo, Inc., Jinju, South Korea was acclimated to the azo dye AB113 (200 mg/L) carried in the dye wastewater obtained from a dye wastewater treatment plant in Daegu City, South Korea. Upon noticeable decolorization, while shaking at 120 rpm at room temperature for 6 days, various microorganisms were isolated on spread plates of tryptic soy agar (TSA). Initially, two dominant bacterial cultures (B-1 and B-2) and two yeast cultures (Y-1 and Y-2) were isolated and further tested for decolorization effectiveness compared with the parental consortium culture itself.

The four different strains were taxonomically identified based on sequencing of the 16S or 26S rRNA genes. The two bacterial cultures (B-1 and B-2) were identified using the 16S rRNA gene sequencing method. The target gene fragments were obtained using a modified colony PCR method in which the primers 27F (5′-AGAGTTTGATCCTGGCTCAG-3′), 1492R (5′-GGTTACCTTGTTACGACTT-3′), 518R, and 785F were used in PCR amplification. The amplified genes were purified using the PCR Product Purification Kit (Qiagen, Boston, MA, USA), and gene sequencing was performed using the Genetic Analyzer 3730xl (Applied Biosystems, Waltham, MA, USA). A homology search for the analyzed sequences within the NCBI Genebank database was conducted, and their phylogenetic positions were identified using the CLUSTAL-W and MEGA6 software. According to the NCBI BLAST Search, B-1 was identified as *Mesorhizobium* sp. NBIMC_P2 (KF040403) with 99% similarity. B-2 was identified as *Sphingomonas melonis* DAPP-PG 224T (KB900605) according to the EzbioCloud database (Chunlab, Seoul, South Korea). The two yeast cultures (Y-1 and Y-2) were identified using the 26S rRNA sequencing method. The primers Y-F (5′-GCATATCAATAAGCGGAGGAAAAG-3′) and Y-R (5′-GGTCCGTGTTTCAAGACG-3′) were used in PCR amplification. The amplification, purification, and homology search processes were performed following the same procedures used for bacterial identification. According to the NCBI BLAST Search, Y-1 and Y-2 were identified as *Apiotrichum mycotoxinivarans* CBS: 10094 (KB900605) and *Meyerozyma guilliermondi* GJ8-2 (KU316708), both with 99% homology (Table 1).

All individual cultures were incubated on TSA, and pure cultures were preserved in phosphate buffer with glycerol (15%) at −80 ℃. Prior to the experiment, frozen cultures were recovered on TSA, and then inoculated into mineral salts medium (MSM) and incubated in a shaking incubator at 27 °C and 150 rpm for 24 h.

### 2.2. Experimental Culture Setup for Biodegradation of the Azo Dye Acid Blue 113

The Acid Blue 113 (CAS No. 3351-05-1) dye used in this study was purchased from Sigma Aldrich, South Korea. The cultures for the biodegradation testing microcosm were set up according to the plan shown in Table 2; each culture carried 100 mL of MSM containing 0.03% (*w/v*) of glucose and 0.0006% (*w/v*) of yeast extract. The MSM had the following composition (g/L): Na_2_HPO_4_, 3.6; (NH_4_)2SO_4_, 1.0; KH_2_PO_4_, 1.0; MgSO_4_, 1.0; Fe(NH_4_) citrate, 0.01; and CaCl_2_.2H_2_O, 0.1, and 10 mL of a trace element solution of the following composition (mg/L) was added to 1 L of the MSM: ZnSO_4_·7H_2_O, 10.0; MnCl_2_·4H_2_O, 3.0; CoCl_2_·6H_2_O, 1.0; NiCl_2_·6H_2_O, 2.0; Na_2_MoO_4_·2H_2_O, 3.0; H_3_BO_3_, 30.0 and CuCl_2_·2H_2_O, 1.0.

After autoclaving the MSM, the stock solution of glucose and yeast extract, which was filter-sterilized using Acrodisc syringe filters (0.2 μm; Pall Laboratory, Westborough, MA, USA), was added to the medium with AB113 dye at different dye concentrations (20, 50, and 100 mg/L). The medium of each culture was inoculated with an aliquot of each of the 24 h grown cultures (5%, *v/v*) and incubated at 27 °C while shaking at 150 rpm. An appropriate amount from each culture was withdrawn for spectrophotometric measurement of growth (at 600 nm) and azo dye biodegradation (at 560 nm).

### 2.3. Monitoring the Cell Growth and Dye Decolorization

For cell growth monitoring, one milliliter of sample from each culture was regularly withdrawn and its growth was monitored at λ_600nm_.

For dye degradation monitoring, samples were withdrawn at regular intervals and centrifuged at 8000× *g* rpm for 15 min at 4 °C. The absorbance of each supernatant was measured at maximum absorbance wavelength, λ_560nm_, using a UV-visible spectrophotometer (Optizen POP, K Lab, Daejeon, Korea). The decolorization efficiency was expressed as in the following equation, as previously described [32]:

(1) $$\mathrm{Decolorization}\;\mathrm{rate}\;\left(\%\right)=\frac{A_{\mathrm{initial}}-A_{\mathrm{final}}}{A_{\mathrm{initial}}}\times 100$$

where A_initial_ and A_final_ represent the initial and final absorbances at λ_560nm_.

### 2.4. RNA Extraction and cDNA Library Sequencing of the Transcriptome

To examine the different gene expression profiles and to perform gene annotation onset of useful genes based on gene ontology pathway information, whole transcriptome sequencing of *Sphingomonas melonis* was performed. The analysis compared the two groups of *S. melonis* B-2 growth depending on the experimental conditions, the control (cultured in absence of azo dye Acid Blue 113 at 50 mg/L) and the treatment (cultured in presence of azo dye Acid Blue 113 at 50 mg/L), using RNA-sequencing. Total RNA from the control and treated samples were isolated and contaminated DNA was eliminated. The Ribo-Zero rRNA Removal Kit (Bacteria: Illumina, San Diego, CA, USA) was used to purify the RNA. The sequencing library was constructed using TruSeq Stranded Total RNA Sample Prep Kit (Illumina, San Diego, CA, USA) according to the manufacturer’s protocol.

### 2.5. De Novo Assembly of the S. melonis B-2 Transcriptome

The purified RNA molecules were randomly fragmented for short-read sequencing and reverse transcribed into cDNA. RNA sequencing and de novo transcriptome assembly were conducted to create reference sequence libraries for *S. melonis* B-2. Then, the sequencing adapter was ligated onto both ends of the cDNA fragments. After amplifying fragments using PCR, fragments with insert sizes between 200–400 bp were selected and sequenced using Illumina’s HiSeq 4000. Raw reads in FASTQ format were firstly processed through FastQC [33]. We trimmed the quality control of the sequenced raw reads using trimmomatic [34]. Overall read quality, total bases, total reads, GC (%) and basic statistics were calculated. Trimmed reads were mapped to the reference genome with Bowtie [35]. Based on transcript length and depth of coverage, expression profiles are represented as read counts and normalization values. In this study, the FPKM (Fragments Per Kilobase of Transcript per Million Mapped Reads) or the RPKM (Reads Per Kilobase of Transcript per Million Mapped Reads) values were used to normalize the read counts.

### 2.6. Analysis of Differential Gene Expression Involved in Azo Dye Degradation

Four independent samples were analyzed and the libraries were generated by sequencing analysis using SAMtools [36]. Gene expression levels of all samples were estimated by HTseq version 0.6.1p1 [37]. The expression values between the control and the treatment were compared according to the RPKM values, with the cutoff of L_2_fc > 1. The topGO package of R was used to perform the GO enrichment analysis and the EggNOG characterization of DEGs was conducted manually. The statistical enrichment of DEGs in KEGG pathways in *S. melonis* B-2 was analyzed.

## 3. Results

### 3.1. Time Courses of Dye Decolorization by Various Cultures

**Figure 1:**
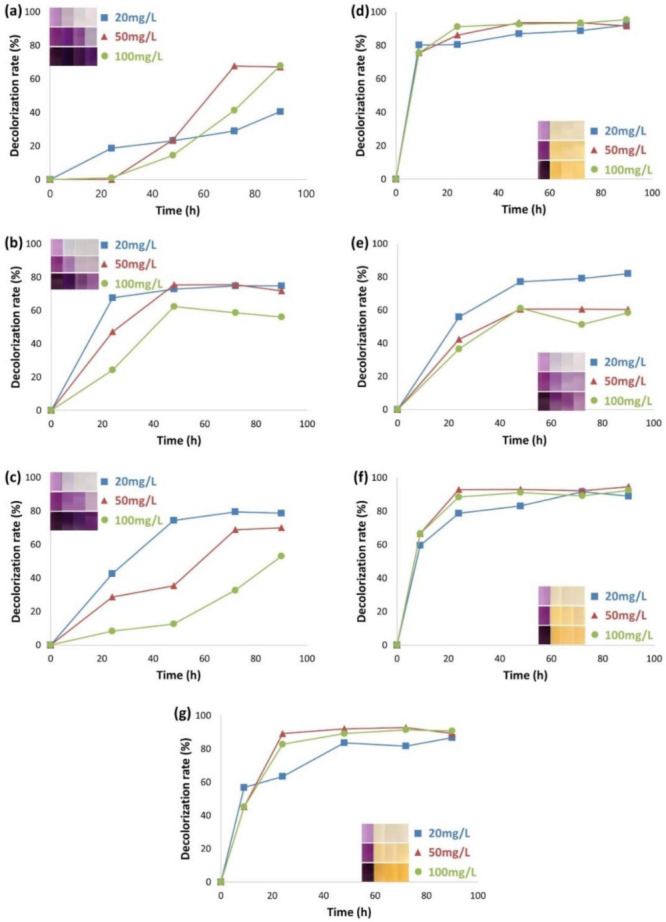
Time course for the azo dye decolorization rates of various pure cultures and consortia of bacteria and yeasts. (**a**) B-1, (**b**) B-2, (**c**) B-C, (**d**) Y-1, (**e**) Y-2, (**f**) Y-C, and (**g**) T-C. The four color boxes show the sample’s color difference over four days. The legend (mg/L) represents different concentrations of the AB113 added.

The time courses for the decolorization rates (%) for all cultures at different concentrations of AB113 dye were shown in Figure 1. Cultures B-1 and B-2 achieved maximum decolorization rates of approximately 67.8% and 75.4% in 50 mg/L dye within 72 h and 48 h, respectively (Figure 1a,b).

The lower decolorization rate for B-1 seemed to be linked to its slow growth, and its maximum decolorization ability appeared after 60 h. In the consortium B-C, the slow growth of B-1 appeared to slow the faster growth of B-2 (50 and 100 mg/L dye), while B-1 did not interfere with the decolorization activity of B-2 at the lower dye concentration (20 mg/L).

As shown in Figure 1d,e, Y-1 decolorized approximately 77.1% in 9 h, while Y-2 achieved a decolorization rate of about 60% in 48 h (50 and 100 mg/L dye), indicating that Y-1 was a faster degrader of the dye. However, when both of the yeast cultures were combined, not much of a synergistic effect was observed in decolorization, and even the speed was decreased. This might be because there was competition among these two cultures for glucose as the reducing agent required for decolorization.

Obviously, the Y-C decolorization rate was much higher than that of the B-C. Yeasts seemed to be less sensitive to the dye concentration gradient, while B-C decolorization was more negatively affected by the higher dye concentration. In general, however, decolorization rates gradually increased over time. The total consortium carrying all four cultures (B-1, B-2, Y-1, and Y-2) achieved more than 80% decolorization after 24 h (50 and 100 mg/L dye) since the slow decolorization of B-C was overcome by the Y-C (Figure 1).

Overall, T-C might be useful in the bioremediation of azo dye materials and their degradative products since the consortium carrying all four members in interaction would be more resilient to fluctuations in environmental conditions (e.g., kinds and amounts of target substrates, salinity, pH, temperature, ORP, etc.) than individual cultures.

In the overall experiment the strain B-2 appears to be a potential bacterial strain for the efficient degradation of AB113 and for the study of RNA-seq analysis due to its available genome for the yeast strain is not available.

### 3.2. Monitoring of Organics and Biodegradation Process by Microbial Consortia for the Azo Dye AB113

**Figure 2:**
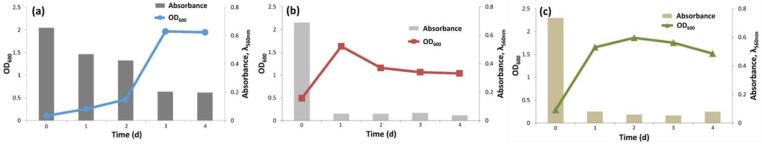
Comparative analysis of consortium cultural growth and concomitant decolorization of the azo dye Acid Blue 113 (50 mg/L) over time. (**a**) B-C, (**b**) Y-C, and (**c**) T-C.

The cell population density (OD_600_) and its concomitant decolorization activity at 560 nm over time were comparatively analyzed. At the beginning (days 1 and 2), the growth of the B-C was much slower than that of the yeast. In the overall consortia, there was a strong negative correlation between OD_600_ and the λ_560nm_ because absorbance at 560 nm decreased as the OD_600_ increased. The B-C had a longer lag phase (2 days), while the Y-C seemed to have a shorter lag period (Figure 2a,b). B-C reached its plateau growth after 3 days, while it took only a day for Y-C to reach its plateau.

There was a good positive relationship between the cell growth and the azo dye degradation rate. Interestingly, T-C maintained its growth level (1.5 OD_600_) after 1 day, probably due to the presence of B-C. This may indicate that the bacteria community could help the yeast community survive under adverse growth conditions.

**Figure 3:**
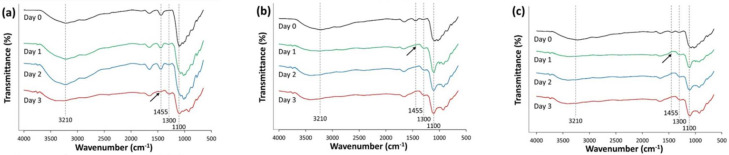
Time course for the FT-IR profiles of the three microbial consortia during biodegradation of the azo dye Acid Blue 113. (**a**) B-C, (**b**) Y-C, and (**c**) T-C.

The collaborative interaction between bacterial and yeast communities may provide a great advantage for the bioremediation of azo dye compounds on a field scale as it has been shown that they can stably maintain a specific consortium that is more resilient to the target degradative compounds, their potentially toxic metabolites, and other nearby hazardous substrates as well as to varying environmental conditions [38]. The FT-IR profiles for the three different consortia during dye biodegradation are shown in Figure 3. FT-IR spectra analysis enables the determination and understanding of the various functional groups involved in the process of biotransformation. The peak at 1455 cm^−1^ indicates the presence of stretching due to azo bonds. In detailing the spectrum, the intense band at 1621 cm^–1^ can be attributed to the bending vibrations of the aromatic ring. Focusing on the region, the stretching vibration of the azo bond is the 3000–2800 cm^–1^ region. The disappearance of this particular peak, therefore, means that decolorization has been achieved. As shown in Figure 3, the arrows may indicate evidence of azo bond cleavage. These results corroborated the previous decolorization results and both results confirm that significant decolorization was achieved after 72 h for B-C and 24 h for both Y-C and T-C. It was also observed that the number of OH or NH groups increased in all the consortia as the intensity of the 3210 cm^−1^ region increased as the dye degraded. Decanedioic acid and propanoic acid possess the OH group in their chemical structures. Again, the transmittance at 3210 cm^−1^ showed a similar trend to that of 1455 cm^−1^ for the azo bond. Moreover, the NH_2_ groups were detected in the degraded samples at the 1300 cm^−1^ wavenumber.

This is evidence of the biodegradation of AB113. These NH_2_ groups are also present in aniline, which were detected as metabolites after decolorization. In addition, the size of the peak at 1100 cm^−1^ representing SO_2_ decreased over time in B-C (Figure 3a) due to bacterial biodegradation. In contrast, the yeast group was not able to degrade this group, while the gradual decrease in this peak was observed in T-C, which carried the B-C (Figure 3c). In terms of secondary metabolites after decolorization, both the bacterial cultures showed an efficient degradative activity at the wave number between 3000–2800 cm^–1^.

### 3.3. Results of Transcriptome Data and Quality Control Evaluation

Comparing two different species present in the consortium, *S. melonis* B-2 was selected as an appropriate candidate for the transcriptome analysis. *S. melonis* B-2 was then grown in the presence of Acid Blue 113 (50 mg/L) (treatment) and the absence of the dye (control). Total RNA was extracted from two groups of the induction experiments. A total of 12.9 GBP clean data were obtained from transcriptome analysis of four samples and 138,562,340 reads were produced.

The average GC content (%) was 62.39% and the percentage of Q30 bases was 94.38% and above. The clean reads of each sample were compared with the designated reference genome, and the alignment efficiency was correct and efficient. The volcano diagram showed that the expression difference compared to the control according to the expression volume was significantly higher. The volume plot was drawn with the volume as an *X*-axis and the log2 fold change as a *Y*-axis. Even though the foldchange might be different by two-fold, the specific genes with higher volume may be more credible.

### 3.4. Gene Expression Pattern Analysis and Clustering of DEGs

**Figure 4:**
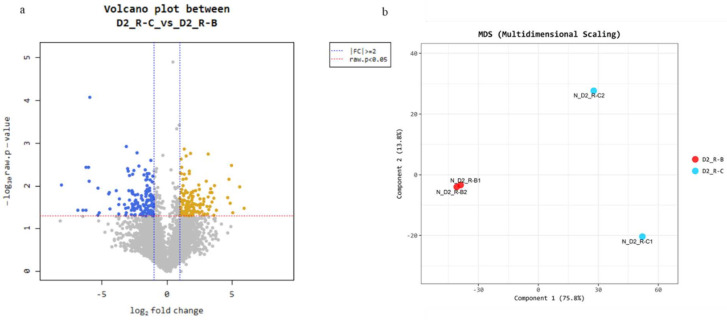
(**a**) Scatter plot shows expression levels between comparison pairs as a scatter plot. The *X*—axis is control and *Y*—axis is the average normalized value of the DEGs group. The colored dot by volume which satisfies the |fc| ≥ 2 and independent *t*-test raw *p*-value < 0.05. (**b**) Multidimensional scaling comparison between control and treatment.

After the read mapping, the DEG analysis was performed on a comparison pair D2_R-C (control) vs. D2_R-B (treatment) using RPKM. Two replicates for each sample were analyzed to find its significance. The result showed 272 significant genes were selected on the conditions of |fc| ≥ 2 and the independent *t*-test raw *p*-value < 0.05. Among the significantly different 272 DEGs, 131 DEGs were up-regulated and 141 DEGs were down-regulated. In other words, 131 DEGs were expressed significantly higher in *S. melonis* B-2 exposed to Acid Blue 113 than that of normal conditions, while 141 DEGs were expressed less (Figure 4a). The identification of outlier samples or the similar expression patterns between the samples were analyzed using multidimensional scaling analysis (MDS). The scattering in component 1 was 75% and shows a higher correlation in the normalized D2_R-B1 and D2_R-B2 as shown in Figure 4b.

### 3.5. Differential Grouping of DEGs in the GO Database Analysis

**Figure 5:**
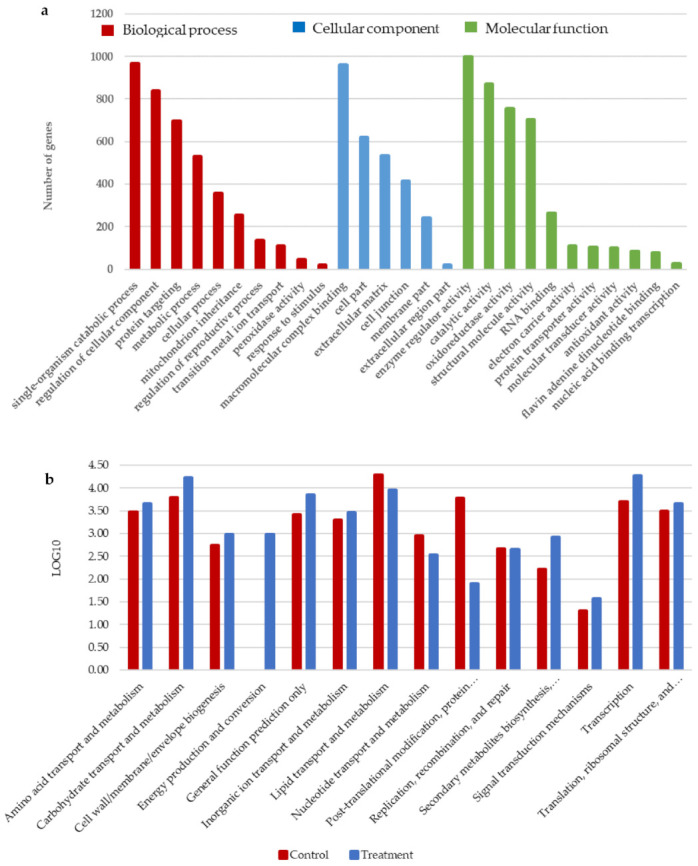
Functional categorization as (**a**) gene ontology (GO) and (**b**) EggNOG of genes differentially expressed under the azo dye Acid Blue 113 induction (treatment) condition.

The GO database was used to classify the annotated UniGenes for the functional annotation of the *S. melonis* B-2 transcriptome (Figure 5a). A total of 12,548 genes were obtained, of which 10,780 genes were annotated, and the annotated efficiency was 86%. The differential grouping of DEG between the control and the treatment were categorized using Gene Ontology. GO enrichment analysis showed that the DGEs are mainly classified into three categories: the biological process (3930 genes), cellular component (2761 genes), and molecular function (4089 genes). Representative categories within the biological process (metabolic process, cellular process, single-organism process, peroxidase activity, etc.), cellular component (cell part, organelle, etc.), and molecular function (binding, catalytic activity, etc.) were visualized. There were 39 subcategories involved in the biological process, including representative subcategories as the single-organism catabolic process (965 UniGenes), regulation of cellular component (836 UniGenes) and protein targeting (698 UniGenes). Macromolecular complex binding (958 UniGenes), cell part (618 UniGenes) and extracellular matrix (531 UniGenes) came under the cellular component category. Finally, enzyme regulator activity (997 UniGenes), catalytic activity (868 UniGenes), oxidoreductase activity (754 UniGenes) and structural molecule activity (703 UniGenes) were categorized under molecular function.

### 3.6. Differential Grouping of DEGs and Their Annotation on EggNOG Database

Annotated UniGenes were mapped to the annotations of corresponding orthologous groups in the EggNOG database to identify enzymes distributed in orthologous groups and non-supervised orthologous groups. (Figure 5b). The RPKM value for the two pairs of the control and the treatment were normalized through LOG10 and their mean was calculated. For EggNOG annotation, 272 significant genes were divided into 14 EggNOG categories. Some of the UniGenes were assigned to more than one category. The largest proportion of UniGenes belonged to “Carbohydrate transport and metabolism”, with a log10 value of 4.22 in the treatment, followed by “Lipid transport and metabolism” with 3.96 and “Amino acid transport and metabolism” and “Translation, ribosomal structure, and biogenesis” with values of 3.66 and 3.65, respectively. The “Energy production and conversion” was only present in the treatment with a Log_10_ value of 3.0. Most of the categories were dominant in the treatment process, while “Lipid transport and metabolism”, “Nucleotide transport and metabolism” and “Post-translational modification, protein turnover, and chaperones” were dominant in the control.

### 3.7. Gene Expression Pattern Analysis and Clustering of DEGs

Based on the GO and EggNOG enrichment analyses, enzymes involved in the degradation of AB113 were selected out of four important enriched GO categories (Table 3). Three DEGs enriched in heme-binding classes encoded cytochrome P450. Oxidoreductase contained NADH quinone oxidoreductase and NADH flavin reductase, encoding six DEGs. The catalytic activity had four enzymes encoded by nine DEGs, and all of them were redox enzymes. The metabolic process contained aromatic ring-hydroxylating dioxygenase with three DEGs. Oxidoreductase activity, metabolic process and most of the genes encoding catalytic activity were upregulated. CYP450 is a key enzyme for the heme-linkage through the cell wall, and it was slightly downregulated compared with the control. Catalase HPII, NADH quinone oxidoreductase and NADH flavin reductase are the key enzymes for the degradation of the azo bond in AB113.

### 3.8. Expression Patterns of Azo Dye Degradative Enzymes and Their Metabolic Pathway Connections in the Degradation

**Figure 6:**
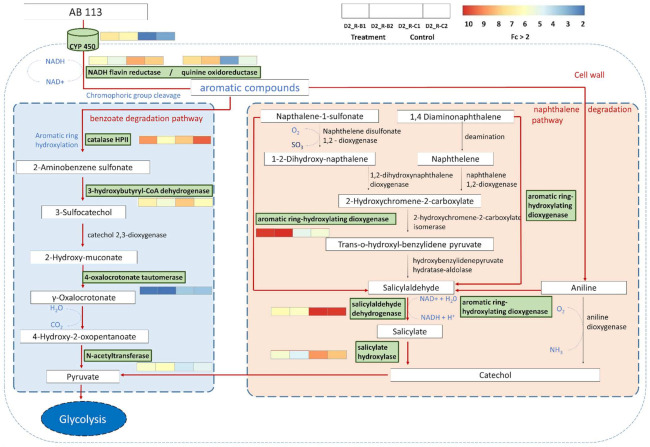
*S. melonis* B-2 DEG transcriptional changes in the AB113 degradation pathway. The enzymes present in the samples were represented in green boxes, whereas the other enzymes are mentioned for reference. The enzyme reactions and the arrows in red color marked between the metabolites represent the directions of catalytic reactions. The expression patterns of control and treatment with fc > 2 values in each enzyme are shown in a heat map.

Enzymes involved in the pathways of azo bonds and aromatic compounds were identified by transcriptome analysis. The degradation of AB113 was generally performed through the benzoate and naphthalene degradation pathways. As shown in Figure 6, the metabolism of azo dyes mainly undergoes a two-step reaction. The key enzyme to catalyze the reaction is the CYP450 enzyme system initiating the heme-linking process. The heme-linking reaction was performed by three CYP450 genes, all of which were downregulated. NADH flavin reductase, quinine oxidoreductase and catalase HPII are the azo bond-degrading enzymes replacing the azo reductase. The azo bond-degrading enzyme contained six upregulated genes and one downregulated gene. The degradation of the azo bond allows 2-aminobenzene sulfonate to be degraded through the benzoate degradation pathway and naphthalene-1-sulfonate, 1,4 diaminonaphthalene and aniline to be degraded through the naphthalene degradation pathway and come under the aromatic degradation pathway. 3-hydroxybutyryl-CoA dehydrogenase, 4-oxalocrotonate tautomerase and N-acetyltransferase lead the degradation of benzene to the pyruvate and end in the glycolysis cycle.

The key enzymes for the degradation of naphthalene and aniline are naphthalene disulfonate 1,2—dioxygenase and aniline dioxygenase. Due to its absence in the *S. melonis* B-2, aromatic ring-hydroxylating dioxygenase was considered as a putative alternative enzyme for the degradation of naphthalene and aniline. The aromatic ring-hydroxylating dioxygenase skips the pathway from Naphthalene-1-sulfonate, 1,4 diaminonaphthalene and aniline to salicylaldehyde. Salicylaldehyde dehydrogenase converts the salicylaldehyde to salicylate through electron transfer and salicylate hydroxylase converts it into catechol. The catechol is converted into pyruvate, and this process leads to the glycolysis.

## 4. Discussion

We identified the key enzymes involved in AB113 degradation using transcriptional sequencing techniques and bioinformatics analysis. For example, NADH flavin reductase, quinine oxidoreductase and CYP450 have been widely reported in dye degradation and aromatic ring-hydroxylating dioxygenase and catalase HPII which has been rarely studied in dye degradation [39,40,41]. We inferred a mechanism of AB113 degradation by these enzymes. These specific enzyme relationships in the azo and aromatic degradation pathway and the role played by an alternative enzyme (NADH flavin reductase, quinine oxidoreductase and CYP450) appeared to be connected in a series of aromatic degradation pathways [39].

In our study, *S. melonis* B-2 reached its maximum range of 85% in 60 h by adding 50 mg/L, and the *Apiotrichum mycotoxinivarans* reached 87% of decolorization within 24 h. The relatively lower decolorization activity of B-1 (20 mg/L dye) seemed to be due to a high concentration of carbon source compared to its initial dye concentration. Previous research reported that in the biological decolorization of azo dyes, microorganisms require a carbon source as they are unable to use the azo compounds as the sole carbon source. Nevertheless, excessive amounts of carbon can reduce the decolorization rate as microorganisms prefer to consume external carbon sources rather than dyes. FT-IR spectra analysis enables the determination and understanding of the various functional groups involved in the process of biotransformation [19,20]. At 1666 cm^–1^, a low intensity occurs, which can be attributed to the sheer vibrations of the amino group or the aromatic C-N bond, demonstrating the presence of the substituted naftanilic ring [42,43]. We identified the key enzymes involved in AB113 degradation using transcriptional sequencing and bioinformatic analysis. For example, NADH flavin reductase, quinine oxidoreductase and CYP450 have been widely studied in dye degradation and aromatic ring-hydroxylating dioxygenase and catalase HPII which has been rarely studied in dye degradation [39,40,41]. These enzymes appeared to be responsible for the reduction of azo bonds in AB113. These specific enzyme relationships in the azo and aromatic degradation pathway and the role played by an alternative enzyme (NADH flavin reductase, quinine oxidoreductase and CYP450) appeared to be connected in a series of aromatic degradation pathways [39].

Under anaerobic conditions, the degradation of azo dyes involves reductive cleavage of azo bonds by azo reductases [40,41]. The process results in dye decolorization and the generation of colorless amines by the transfer of electrons from electron donors to acceptors [42]. In this study, 2-aminobenzene sulfonate appeared to be degraded into pyruvate by *Sphingomonas melonis* B-2. According to the previous studies, 4-aminobenzene sulfonate (4-ABS) could be utilized as sole carbon, nitrogen and sulfur source by a co-culture consisting of *Hydrogenophaga* sp. PBC and *Ralstonia* sp. PBA, isolated from textile wastewater treatment plant [43]. *Sphingomonas xenophaga* BN6 can produce redox mediators which significantly increase its ability to reduce azo dyes through aerobic degradation of naphthalene-2-sulfonate (2NS) [44]. A purified form of the selected enzymes is able to decolorize dyes of different chemical structures. It has been shown that enzymes such The enzymes have a similar activity to azoreductase as NADH flavin reductase and quinine oxidoreductase can degrade different types of dyes [45].

In our study, the activity of NADH quinone oxidoreductase, N-acetyltransferase and aromatic ring-hydroxylating dioxygenase increased significantly after the treatment of AB113. According to the research presented in this paper, azo dyes are degraded by bacteria via a reduction reaction in which aromatic ring-hydroxylating dioxygenases catalyze the catalytic cracking of conjugated dye bonds. The NADH quinone oxidoreductase and NADH flavin reductase belong to oxidoreductase [44]. These enzymes have functional similarities to azoreductase. It can be inferred that oxidative reductases such as the NADH quinone oxidoreductase and NADH flavin reductase of *S. melonis* B-2 may be involved in the catalytic cleavage of coupled dye bonds. Morrison et al. (2012) also report the characterization of an anaerobic azoreductase enzyme in *Clostridium perfringens* [45,46]. NADH and FAD, cofactors of the enzyme, provided a peak in its activity. It was discovered that the azoreductase gene corresponded to either FMN Reductase or flavodoxin-2. There have been very few studies reporting flavin-free azo reductases and two monomeric flavin-free azoreductases from *X. azovorans* KF46F [47] and *P. kullae* K24 [48,49] have been described. The significant homologies with flavin-dependent azoreductases and both types of reductases might be replaced by NADH Flavin-reductase and quinine oxidoreductase.

In the present study, we examined the gene transcriptional changes of *S. melonis* B-2 and azo-degrading metabolites at different processing times. *Sphingomonas* species are by far one of the most important dye degrading organisms described to date [50]. The phylogenetic diversity of *Sphingomonas* species in polluted soils has recently been investigated [51]. According to some studies, the slower degradation rates in bacteria exposed to two or more PAHs may be caused by more toxic effects of more water-soluble aromatic hydrocarbons, such as naphthalene, and accumulation of metabolites [52,53,54]. *Pseudomonads* that degrade naphthalene produce nahG, a flavoprotein monooxygenase, which accelerates the conversion of salicylate to catechol [55]. Three strains of *Sphingomonas* sp. did not express a nahG-like gene associated with PAH-catabolic genes. suggested other gene products might have caused the reaction. The salicylate 1-hydroxylase genes were found in three sets within the main catabolic gene region of *Sphingobium* sp. [56]. Monooxygenase in strain P2 may be replaced by these enzymes, and similar genes in other *Sphingomonas* species may also be implicated [50].

Anthranilate, Benzene, bi-phenyl, m-cresol, etc., as well as several enzymes vital for the degradation of the other twenty additional aromatic compounds, such as aniline, naphthalene, xylene, phthalate, and anthranilate, are not detected in B-2. There were genes encoding for aromatic compound transporters in all genomes. As before, aromatic ring-hydroxylating dioxygenase possessed the most aromatic transporters among all the enzymes, which suggests that it might play a role in the oxidative catabolism of aromatic amines as a consequence of reductive cleavage of the azo bond in azo dye molecules [57]. Salicylate hydroxylase and salicylate-1-hydroxylase, described as enzymes, and homologous proteins reported in related *Sphingomonas* viz. strains are the components transforming salicylic acid to catechol [58,59]. In addition, catalase may convert benzoic acid and p-toluic acid to catechol and 4-methyl catechol [60].

## 5. Conclusions

In this study, we successfully selected a bacterium (*S. melonis* B-2) for the efficient dye decolorization of the azo dyes, which are a major product of dye production. More than 80% of decolorization within 24 h (50 and 100 mg/L dye) was achieved by *S. melonis* B-2. There was a good positive relationship between the cell growth and the azo dye degradation rate, which can also support the mineralization potential. It was concluded that all the data of decolorization, cell growth and FT-IR spectra collectively provided consistent evidence for the azo dye decolorization and potential mineralization of the dye by the stably maintained culture. Furthermore, RNA-transcriptome analysis showed that there was a significant difference in the gene expression of *S. melonis* B-2 when exposed to azo dye Acid Blue 113. The up-regulated genes encoding oxidoreductases in the AB113 environment indicate that they are the key enzymes for AB113 azo bond cleavage. The occurrence of benzoate and naphthalene degrading pathways indicated their involvement in the downstream pathways of azo dye AB113 degradation. This study explored the process of AB113 biodegradation at the molecular and biochemical levels and provided a theoretical basis for its practical application. It will be a good opportunity to use these DEGs encoding specific enzymes as potential biomarkers in the bioremediation process of the azo dye wastewater treatment at the industrial scale. In future studies, we aim to apply these potential biomarkers in monitoring the dye wastewater treatment at a full-scale level (e.g., using a real-time transcriptase PCR system and metatranscriptomic analysis).

## Figures and Tables

**Table 1:** Identification of microorganisms efficient for the biodegradation of azo dye Acid Blue 113.

Strain Name	Identification	Best Match (Closest Strain)					
		Name	Strain	Authors	Accession	Similarity (%)	Mismatch/Total nt
B-1	*Mesorhisobium* sp.	AVBN_s (*Mesorhisobium* sp.)	NBIMC_P2-C1	Lo, S.C et al., 2014	KF040403, AVBN01000018	100	0/1406
B-3		*Mesorhisobium haukuii*	IAM 14158	(Chen et al., 1991) Jarvis et al., 1997	D12797	98.42	22/1399
B-2	*Sphingomonas* *melonis* B-2	*Sphingomonas* *melonis*	DAPP-PG 224	Buonaurio at al., 2002	KB900605	100	0/1409
Y-1	*Apiotrichum* *mycotoxinivorans*	*Apiotrichum* *mycotoxinivorans*	CBS: 10094	Vu, D at al., 2016	KY109958	99	2/603
Y-2	*Meyerozyma guilliermondi*	*Meyerozyma* *guilliermondi*	Gj8-2	Han, S. 2016	KU316708	99	1/578

**Table 2:** Experimental setup of microbial cultures for the biodegradation of azo dye Acid Blue 113.

Cultures *	Species of Cultures Grown in Culture	Inoculation Amount (%)	Applied Conc. of AB 113 (mg L^−1^)
B-1	*Mesorhizobium* sp.	5	20, 50, 100
B-2	*Sphingomonas melonis*	5	20, 50, 100
Y-1	*Apiotrichum mycotoxinivarans*	5	20, 50, 100
Y-2	*Meyerozyma guillermondi*	5	20, 50, 100
B-C ^a^	*Mesorhizobium* sp.	2.5	20, 50, 100
	*Sphingomonas melonis*	2.5	
Y-C ^b^	*Apiotrichum mycotoxinivarans*	2.5	20, 50, 100
	*Meyerozyma guillermondi*	2.5	
T-C ^c^	*Mesorhizobium* sp.	1.25	20, 50, 100
	*Sphingomonas melonis*	1.25	
	*Apiotrichum mycotoxinivarans*	1.25	
	*Meyerozyma guillermondi*	1.25	

* Each culture contains 100 mL of mineral salts medium (MSM) (see the details in Section 2.2) in an Erlenmeyer flask (250 mL) together with 0.03% glucose and 0.0006% yeast extract, incubated at 27 °C for 4 days in a shaking incubator (150 rpm). ^a^ Bacteria consortium, ^b^ Yeast consortium, ^c^ Total microbial consortium.

**Table 3:** Clustering of DEGs and their gene expression patterns.

GO Term	Enzymes	Gene Id	Up-Regulated	Coverage (%)	E-Value
Catalytic activity	catalase HPII	gene2971	4.99	98.13	0
Oxidoreductase activity	NADH quinone oxidoreductase	gene2085	5.38	94	7 × 10^−1^
		gene2088	8.30	95.68	1 × 10^−8^
		gene2090	4.88	96.57	5 × 10^−3^
		gene2095	10.52	98.04	8 × 10^−3^
		gene2099	11.64	89.64	0
Catalytic activity	N-acetyltransferase	gene285	8.83	84.88	5 × 10^−1^
		gene680	2.06	94.4	9 × 10^−4^
		gene990	2.31	94.66	0
		gene1312	3.11	84.75	2 × 10^−6^
		gene1850	3.84	99.31	0
		gene2426	5.32	99.55	0
Metabolic process	aromatic ring-hydroxylating dioxygenase	gene1111	10.69	98.12	5 × 10^−5^
		gene2173	11.85	87.36	1 × 10^−9^
		gene2473	8.88	85.01	3 × 10^−1^
**GO Term**	**Enzymes**	**Gene Id**	**Down-Regulated**	**Coverage**	**E-Value**
Heme binding	cytochrome P450	gene3695	3.17	35.58	8 × 10^−1^
		gene3489	2.10	98.29	6 × 10^−6^
		gene1577	3.44	96.36	1 × 10^−1^
Oxidoreductase activity	NADH flavin reductase	gene1341	2.89	99.52	3 × 10^−7^
Catalytic activity	3-hydroxybutyryl-CoA dehydrogenase	gene953	2.48	93.1	2 × 10^−9^
	salicylate hydroxylase	gene3343	2.86	49.36	0.0003
	salicylaldehyde dehydrogenase	gene420	3.56	96.61	8 × 10^−7^

## Data Availability

Not applicable.
